# SarcTrack: An Adaptable Software Tool for Efficient Large-Scale Analysis of Sarcomere Function in hiPSC-Cardiomyocytes

**DOI:** 10.1161/CIRCRESAHA.118.314505

**Published:** 2019-04-11

**Authors:** Christopher N. Toepfer, Arun Sharma, Marcelo Cicconet, Amanda C. Garfinkel, Michael Mücke, Meraj Neyazi, Jon A.L. Willcox, Radhika Agarwal, Manuel Schmid, Jyoti Rao, Jourdan Ewoldt, Olivier Pourquié, Anant Chopra, Christopher S. Chen, Jonathan G. Seidman, Christine E. Seidman

**Affiliations:** 1 From the Department of Genetics (C.N.T., A.S., A.C.G., M.N., J.A.L.W., R.A., M.S., J.R., O.P., J.G.S., C.E.S.), Harvard Medical School, Boston, MA; 2 Image and Data Analysis Core (M.C.), Harvard Medical School, Boston, MA; 3 Cardiovascular Medicine, Radcliffe Department of Medicine (C.N.T.), University of Oxford, United Kingdom; 4 Wellcome Centre for Human Genetics (C.N.T.), University of Oxford, United Kingdom; 5 Cardiovascular and Metabolic Sciences, Max Delbrück Center for Molecular Medicine, Berlin, Germany (M.M.); 6 German Centre for Cardiovascular Research, Berlin, Germany (M.M.); 7 Charité-Universitätsmedizin, Berlin, Germany (M.M.); 8 Hannover Medical School, Germany (M.N.); 9 Department of Pathology (J.R., O.P.), Brigham and Women’s Hospital, Boston, MA; 10 Cardiovascular Division, Department of Medicine (C.E.S.), Brigham and Women’s Hospital, Boston, MA; 11 Deutsches Herzzentrum München, Technische Universität München, Germany (M.S.); 12 Harvard Stem Cell Institute, Boston, MA (J.R., O.P.); 13 Biomedical Engineering, Boston University, MA (J.E., A.C., C.S.C.); 14 The Wyss Institute for Biologically Inspired Engineering at Harvard University, Boston, MA (J.E., A.C., C.S.C.); 15 Howard Hughes Medical Institute, Chevy Chase, MD (C.E.S.).

**Keywords:** cardiomyocyte contractility, cell imaging, hypertrophic cardiomyopathy, induced pluripotent stem cells, MYK-461, myosin binding protein-C, sarcomeres

## Abstract

**Rationale:**

Human induced pluripotent stem cell–derived cardiomyocytes (hiPSC-CMs) in combination with CRISPR/Cas9 genome editing provide unparalleled opportunities to study cardiac biology and disease. However, sarcomeres, the fundamental units of myocyte contraction, are immature and nonlinear in hiPSC-CMs, which technically challenge accurate functional interrogation of contractile parameters in beating cells. Furthermore, existing analysis methods are relatively low-throughput, indirectly assess contractility, or only assess well-aligned sarcomeres found in mature cardiac tissues.

**Objective:**

We aimed to develop an analysis platform that directly, rapidly, and automatically tracks sarcomeres in beating cardiomyocytes. The platform should assess sarcomere content, contraction and relaxation parameters, and beat rate.

**Methods and Results:**

We developed SarcTrack, a MatLab software that monitors fluorescently tagged sarcomeres in hiPSC-CMs. The algorithm determines sarcomere content, sarcomere length, and returns rates of sarcomere contraction and relaxation. By rapid measurement of hundreds of sarcomeres in each hiPSC-CM, SarcTrack provides large data sets for robust statistical analyses of multiple contractile parameters. We validated SarcTrack by analyzing drug-treated hiPSC-CMs, confirming the contractility effects of compounds that directly activate (CK-1827452) or inhibit (MYK-461) myosin molecules or indirectly alter contractility (verapamil and propranolol). SarcTrack analysis of hiPSC-CMs carrying a heterozygous truncation variant in the myosin-binding protein C (*MYBPC3*) gene, which causes hypertrophic cardiomyopathy, recapitulated seminal disease phenotypes including cardiac hypercontractility and diminished relaxation, abnormalities that normalized with MYK-461 treatment.

**Conclusions:**

SarcTrack provides a direct and efficient method to quantitatively assess sarcomere function. By improving existing contractility analysis methods and overcoming technical challenges associated with functional evaluation of hiPSC-CMs, SarcTrack enhances translational prospects for sarcomere-regulating therapeutics and accelerates interrogation of human cardiac genetic variants.

## Abstract 2

Supplemental Digital Content is available in the text.

Recent technical breakthroughs in pluripotent stem cell technologies^1^ and CRISPR/Cas9 genome editing^2^ have enabled the production of human cardiomyocytes from induced pluripotent stem cells (human induced pluripotent stem cell–derived cardiomyocytes [hiPSC-CMs]) with patient-specific genotypes. Unlike primary cultures of human cardiomyocytes that are difficult to obtain and maintain, hiPSC-CMs can be mass-produced using protocols that are optimized for 2-dimensional or 3-dimensional differentiation.^3^ Moreover, CRISPR/Cas9 genome editing of hiPSC lines with isogenic backgrounds ensures that an observed downstream phenotype reflects the variant under study. Although hiPSC-CMs are often genetically, structurally, and metabolically immature in comparison to endogenous adult cardiomyocytes, evolving methods to pattern^4^ and improve maturation^5^ continue to advance the utility of hiPSC-CMs. Studies of hiPSC-CMs have already contributed insights into cardiomyocyte biology,^6^ heart disease mechanisms,^7,8^ and have uncovered off-target cardiotoxic drug effects.^9,10^

**Editorial, see p 1146**

**In This Issue, see p 1141**

**Meet the First Author, see p 1142**

A critical component of functional analyses of cardiomyocytes is the assessment of dynamic contraction in beating cells. Existing methods assess contraction by imaging cardiomyocyte movement using edge detection, changes in pixel intensities across image frames,^11,12^ or, optical flow-based displacement.^13^ Other methods directly monitor changes in sarcomere length (SL) by fast Fourier transform (FFT)^14^ analysis, 2-photon microscopy,^15^ user-selected sarcomeres that are tagged by fluorescence^16^ or quantum dots conjugated to antibodies that target sarcomere proteins.^17^ These approaches vary with regards to user input, requirements for specialized equipment, and throughput speed, but most output average signals, often using FFT analyses, and are most often applied to well-ordered sarcomeres. Averaging FFT signals is most appropriate for evaluating homogenous populations of cardiomyocytes with linearly aligned sarcomeres that consistently and equally contract. However, the application of these methodologies to study hiPSC-CMs is challenged by their circular morphology and heterogeneity in differentiation and maturation. The orientation of sarcomeres in hiPSC-CMs is variable and contraction is nonlinear, which results in significant cell-to-cell variability.^9^ Customized strategies to pattern hiPSC-CMs addresses some of these issues,^18^ but these platforms add other variables (eg, matrix stiffness) that can impact contractility and also delay experimental throughput.^4^

To address these hurdles, we developed SarcTrack, a rapid, easily implemented MatLab algorithm that directly monitors absolute sarcomere count and dynamic changes in SL, providing parameters for sarcomere content, percent contraction, sarcomere contraction and relaxation durations, and cellular beat rate (Figure 1). These measures are obtained directly from videos of fluorescently labeled Z-disc or M-line pairs (Online Figure I) within each sarcomere and define the mechanics of contraction at the level of the single sarcomere. The analysis of hundreds of sarcomeres per cell provides enhanced statistical power for evaluating contractility across the full cardiac cycle. Using fluorescent tracking of sarcomeres using SarcTrack, we gained direct insight into pharmacological effects on individual sarcomere function that can be extended to subpopulations of sarcomeres within a cell. By contrast, techniques using bright-field microscopy are limited in providing averaged cellular contractile function.^11,12^

**Figure 1.**
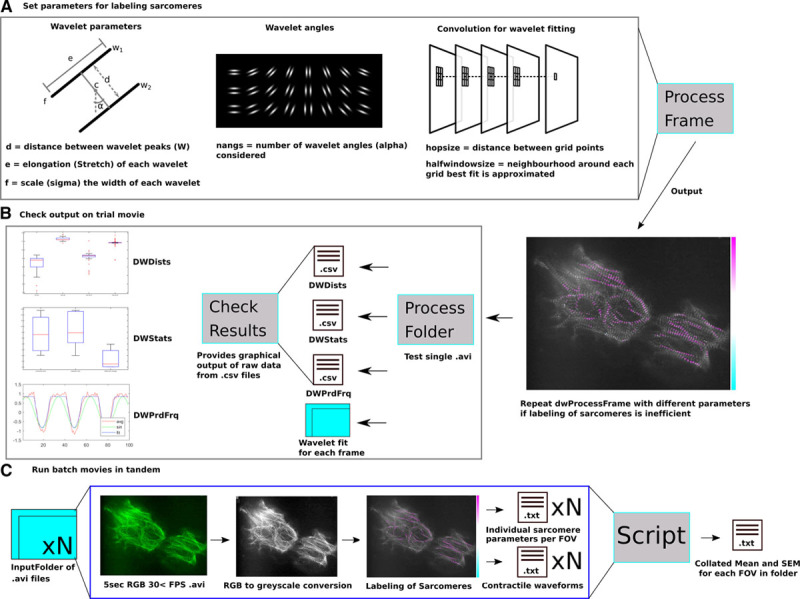
**Using SarcTrack to identify fluorescent sarcomeres.** **A**, Initial parameters are defined on a single frame of an acquired image stack to test fitting of Z-discs by SarcTrack wavelets. Wavelet parameters include the wavelet dimensions (*d*, *e*, *f*), the *df* for fitting the wavelet in certain orientations (*α*, the angles around *c*), and the definition of the grid points when performing the convolution for fitting the wavelets to fluorescent Z-disc pairs (hopsize and halfwindowsize). Once parameters are defined (see Online Methods), dwProcessFrame can be run to visualize the goodness of wavelet fitting to a specific frame. This process is repeated to find the best parameters for labeling Z-discs. In this example, we show a highly disordered cell with significant out of focus fluorescence to demonstrate the ability of these parameters to be adapted to robustly define true Z-disc pairs. **B**, Once parameters have been defined and tested on a single frame a stack can be analyzed using dwProcessFolder. This process allows the user to deposit one stack into a folder for analysis and this provides 4 outputs. A folder containing each individual frame fit with marked wavelets to check fidelity of wavelet fitting, DWPrdFrq providing the period and frequency of beating defined by sinusoidal fitting of the contractile functions, DWDists providing the distance (*d*, in pixels) of each wavelet pair for each frame of the stack. DWStats provides the precalibrated raw values derived from the sawtooth fitting of contractile cycles of each sarcomere tracked including contraction time (frames), relaxation time (frames), the minimum distance between wavelets, and the maximum distance between wavelets (pixels). dwCheckResults will output graphs of these parameters and show the fitting of the sinusoid and sawtooth for the combined sarcomere contractility of the entire field of view (FOV). **C**, Once parameters have been set and quality control of data has been approved using dwCheckResults one can run a folder of multiple movies. A computing cluster can be used to run many movies in parallel, and by using 4 cores, users can analyze 5-second movies every 40 min with parallel batches of up to 100 movies at a time. Once movies are batch analyzed, a script is applied to convert the outputs from DWStats to microns and seconds from pixels and frames. A mean and SEM is obtained from each DWStats file and data is output into a single .txt file. This script also adds the number of sarcomeres tracked in each movie as a separate column to all other parametric means and errors. This workflow ensures that the user can efficiently troubleshoot wavelet fitting and batch analyze hundreds of movies and tens of thousands of sarcomeres in a few hours.

## Methods

The data that support the findings of this study are available from the corresponding authors on reasonable request.

### TTN-GFP and MYOM1-RFP Fluorescent Sarcomere Reporter hiPSC Lines and Differentiation Into hiPSC-CMs

SarcTrack analyzes hiPSC-CMs with fluorescent tags on sarcomere proteins. We previously reported protocols to introduce GFP (green fluorescent protein) onto the Z-disc protein titin (TTN-GFP) in hiPSCs.^19^ Paralleling this approach, we used CRISPR/Cas9-mediated homology-directed repair of hiPSCs to introduce RFP (red fluorescent protein) onto the carboxyl terminus of myomesin-1 (MYOM1-RFP), an M-band protein (Online Figures I through III). Appropriate targeting was confirmed by DNA sequencing. Undifferentiated tagged hiPSCs lack fluorescence, but on differentiation into hiPSC-CMs,^3^ sarcomeres fluoresce, from which sarcomere periodicity can be assessed.^20^

For SarcTrack analyses, fluorescent-tagged hiPSC-CMs (<20-day postdifferentiation) were replated onto imaging-optimized 12-well plates (or 96-well plate for drug treatments, MatTek) containing 1:100 Matrigel (Corning) in RPMI 1640 medium. Two days after replating, cells were returned to RPMI 1640 containing B27 supplement, with insulin, which is changed every 2 days. Imaging was performed at day 30 postdifferentiation of hiPSC-CMs, a commonly used time point for functional analyses.^10^

### Infection of hiPSC-CMs With mApple-ACTN-2 Lentivirus

SarcTrack can assess hiPSC-CMs with fluorescent tags that are delivered by lentivirus (5 μL of viral stock in 2 mL of RPMI+B27+Insulin, to achieve a viral multiplicity of infection ≥1), such as mApple-ACTN-2 lentivirus.^6^ After 24 hours of incubation, infected cells were washed with PBS and replaced with fresh RPMI 1640+B27+Insulin medium. The mApple-ACTN-2 signal was apparent within 2 to 3 days of adding virus.

### Generation and Functional Analysis of Heterozygous *MYBPC3* Loss-of-Function hiPSC-CMs

Using CRISPR/Cas9 editing, we used a guide RNA^21^ to introduce a frameshift variant into exon-2 of the *MYBPC3* gene in TTN-GFP hiPSCs. Subclone analyses yielded an 8-nucleotide deletion in one allele, which was confirmed by DNA sequencing. Mutant *MYBPC3* lines readily differentiated into hiPSC-CMs^3^ and are denoted MyBPC^t/+^ (myosin binding protein-C) TTN-GFP hiPSC-CMs. Control TTN-GFP hiPSC-CM line underwent nucleofection without guides and maintained normal, biallelic *MYBPC3* expression.

### Small Molecules and Drug Treatment on hiPSC-CMs

Small molecules (verapamil and propranolol obtained from Sigma Aldrich, MYK-461 from Cayman Chemical, and CK-1827452 from Selleck Chemicals) were dissolved in water or DMSO (10 mmol/L) and stored at −80°C. hiPSC-CMs (day 30 postdifferentiation) were treated in 96-well imaging plates with compounds diluted in RPMI 1640 plus B27 and insulin medium to achieve half-log doses ranging from 0 to 10 μmol/L final concentrations unless otherwise specified. When sequential increasing doses of a compound (200 μL) was delivered to 96-well imaging plates containing hiPSC-CMs, plates were equilibrated at 37°C and 5% CO_2_ for 1 hour before imaging.

### Traction Force Microscopy on Hydrogels

Micropatterned fibronectin polyacrylamide hydrogel substrates were fabricated as described.^4^ Polyacrylamide gels of 7.9 KPa stiffness were made by adjusting the concentrations of acrylamide and bisacrylamide stock solution (Bio-Rad Laboratories, Hercules, CA), as described.^22^

The contractile forces exerted by hiPSC-CMs on the gel substrates were computed by measuring the displacement of fluorescent beads embedded within the gel as described.^6^ hiPSC-CMs were electrically paced at 1 Hz, and videos of bead motion near the substrate surface, distributed in and around the contact region of a single cell, were acquired at a frame rate of 30 frames per second. The stress vector fields were generated using an open source package of Fiji plugins^23^ and were interpolated with a custom MatLab script. Root mean squares of the magnitudes of single cell stress vectors were computed using the formula:

Root mean squares of the magnitudes of single cell stress vectors=√1(|*S*1|2+|*S*2|2+…+ |*Sn*|2), in which Sn is a single stress vector. Root mean squares of the magnitudes of single cell stress vectors were plotted over time and smoothed using a custom MatLab script. Contractile stress was calculated as the difference between local maximum and minimum root mean squares of the magnitudes of single cell stress vectors and was averaged over 3 to 4 beat periods for each cell.

### hiPSC-CM Video Acquisition and Data Processing

Five-second video imaging was conducted on small hiPSC-CM clusters of 2 to 4 cells maintained at 32°C to 37°C and 5% CO_2_, using a 100X objective of a fluorescent microscope with a minimum acquisition rate of 30 frames per second. Pacing, where specified, was conducted at 1 Hz using a manual electrode. For downstream evaluation of contractility videos, SarcTrack was run on the Harvard Medical School high-performance computing cluster. Data processing required on average 4 CPU cores to analyze a 5-second video in 15 to 20 minutes.

### Mathematical Foundations for SarcTrack-Based Sarcomere Tracking

The SarcTrack algorithm (provided on GitHub at https://github.com/HMS-IDAC/SarcTrack) modeled each sarcomere as a double wavelet, that is, a pair of parallel Morlet wavelets, w_1_ and w_2_ (Figure 1A), with distance *d* between wavelet peaks (eg, peak fluorescence intensity of the sarcomere), center of mass *c* (eg, the midpoint between wavelet peaks), and α, the rotation of the wavelet pair around *c*. The user specifies these parameters, as detailed in Online Methods. A single wavelet was defined as 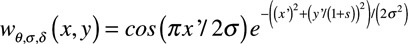, where 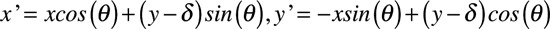. Here 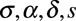 corresponded to scale, angle (in radians), displacement (offset from zero), and stretch of the wavelet, respectively.

We then defined a double wavelet as 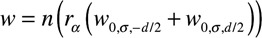, where *r* was a rotation by angle *α* and *n* was a normalization that ensures the double wavelet has mean *0* and norm *1*. This resulted in simply adding 2 single parallel wavelets at distance *d*, rotating the result by an angle *α*, then normalizing.

Each video frame was convolved with a bank of double-wavelet kernels, including kernels for a wide and dense enough set of distances *d*, and a dense enough set of angles *α*. The range of distances was determined by measuring the distances between Z-discs on several frames. The density of distances and angles was determined empirically, based on visual comparison between images and detection outputs. The stretch (elongation) and scale of each individual Morlet wavelet were estimated by analyzing z-disc cross-sections, and the detection outputs.

The bank of double wavelets was given by 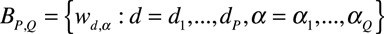, where *P,Q* were the number of different distances and angles, respectively. After convolving a frame *F* with each wavelet in *B*_*P*,*Q*_, the results were a stack of images 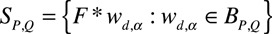, where * was the convolution operator.

After convolutions, a subgrid of pixels was defined. For each pixel in the subgrid, the maximum filtering output was located in a small neighborhood of the pixel, and the angle and distance of the double wavelet of maximum output were recorded. That is, for a location *p*, in the subgrid, the optimal distance *d* and angle *α* were computed as 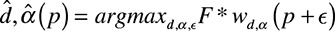 for 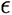 in a small neighborhood or zero. This neighborhood should be small enough so that 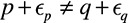 for all 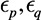 in the neighborhood and *p,q* adjacent grid points (Figure 1A; convolution of wavelet fitting).

After this step, we had a set of pixel locations, with corresponding angles, distances, and magnitudes of best-fitting double wavelets. Although for SarcTrack analyses, we are particularly interested in distances, the angles and magnitudes were useful for reconstructing the image to verify proper fitting. Intuitively, each point of the subgrid should contain a double-wavelet structure that is allowed to snap into the nearest sarcomere by changing *d*, *c*, and *α* until a good fit was reached (Figure 1A; convolution of wavelet fitting). This strategy reasonably assumed continuity in both the time in which neighboring sarcomeres contract, and the distance between their Z-discs. This model allowed us to probe for local distance between Z-discs over subsequent frames created by process folder and output into .csv files (Figure 1B).

After detecting sarcomeres and removing those of low magnitude, visualization of the curve of average distances using CheckResults showed a sawtooth periodic function, akin to adult cardiomyocyte contraction (Figure 1B). Because we were not only interested in maximum and minimum distances between Z-discs, but also contraction and relaxation times, we fitted this curve with a custom-designed periodic curve that was constant for areas corresponding to a relaxed sarcomere and had a nonlinear sawtooth-shaped valley for contraction and relaxation intervals.


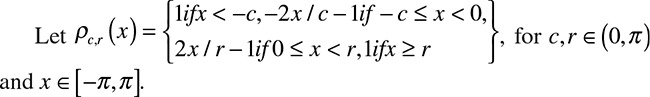


The periodic curve we fitted to the tracked distances of each sarcomere was defined as 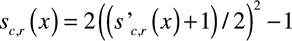, where 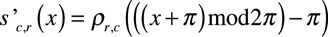.

Fitting was performed by optimizing the correlation between the raw distance data 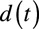 and 
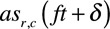
, where *t* is the time parameter (or frame index), *f* is the frequency, *a* is the amplitude, and *δ* the offset. Only parameters *c* (contraction), *r* (relaxation), and *δ* were optimized for each sarcomere independently. Parameters *f* and *a* were precomputed by estimating the period/frequency of the averages of *d(t*) using a simpler model that fits a sinusoidal function.

To initialize the optimization algorithm that fitted the average-distance curve, we first estimated the curve’s period/frequency using a simpler model that fits a sinusoidal function (Figure 1B). This gave an initial approximation for the contraction and relaxation times. However, because sarcomeres do not contract and relax in synchrony, the averaging process overestimated those times. Thus, we independently fitted each curve of selected sarcomeres, using the average fit parameters as starting values in the optimization routine.

Finally, we used a measure of goodness of fit (root mean square error) between raw curves and fitted models to further select sarcomeres and improve the statistical robustness of reported results. To the best of our knowledge, the independent measurement of contraction/relaxation times per sarcomere has not been done, and previous methods have not taken account for asynchronous oscillations in sarcomeres.

Once these results were checked for fidelity, we applied these tested parameters for sarcomere tracking in multiple movies (Figure 1C). By performing tandem analyses using a computer cluster (or computer with multiple processor cores), movies were simultaneously analyzed. Once completed, we applied a custom script to collate the mean and SEM^24^ for each analyzed field of view from a movie, which was reported as an individual row in a single .txt file (Figure 1C).

### Production of Synthetic Sarcomeres for SarcTrack Benchmarking

The algorithm for generating synthetic sarcomeres is deposited online (https://github.com/HMS-IDAC/SarcTrack). Synthetic movies were generated by placing parallel single wavelets at predefined distances. We generated movies with 5 lines of 9 wavelets each.

The distances between wavelets changed over time according to the sawtooth function defined in the methods for SarcTrack. Half of the synthetic sarcomeres contraction cycle was delayed to test how the algorithm coped with asynchronous contraction cycles. Because we knew the parameters that generate the distances (eg, minimum and maximum distance between wavelets, contraction, and relaxation times), we confirmed that the algorithm properly identifies synthetic sarcomeres.

### Statistics

Where single statistical comparisons were applied, a Student *t* test was used with a significance cutoff of *P*<0.05. Where multiple comparisons were made, ANOVA was used with multiple comparisons tested by post hoc Bonferroni correction with a statistical significance cutoff of *P*<0.05. All significances are stated in the text with the corresponding *P* value. All data are stated as mean with SEMs.^24^

## Results

SarcTrack was designed to image (Online Figure I) fluorescently labeled sarcomeres in hiPSC lines^19,20^ such as lines with endogenous GFP-tags on titin (TTN-GFP; Online Figure II) or myomesin (MYOM1-RFP; Online Figure III). Cells can be exogenously labeled, using lentivirus with cargo such as ACTN-2 labeled with mApple (Online Figure IB).^6^ hiPSCs with fluorescently tagged sarcomere genes exhibited no alterations in pluripotency in comparison to untagged isogenic hiPSCs (Online Figure IV). On differentiation into hiPSC-CMs fluorescence microscopy (100X oil immersion objective) showed localized fluorescence at specific sarcomere domains: Z-discs tagged by TTN-GFP and ACTN-2-mApple labeled Z-discs and M-band tagged by MYOM1-RFP (Online Figure IB). An illustration of how contraction and relaxation times were delineated, as well as representative traces of tracked sarcomeres in paced and unpaced states are shown in Online Figure IC and ID. Differentiation of the TTN-GFP reporter hiPSC line into skeletal muscle fibers^25–27^ (Online Figure V), demonstrated the utility of these lines to label skeletal muscle sarcomeres for SarcTrack analyses.

SarcTrack employs a MatLab algorithm (see Methods and Online Methods) that fitted individual sarcomeres as a pair of parallel Morlet wavelets (w_1_ and w_2_) with distance *d* between crests, center of mass *c*, and orientation *α* (Figure 1A). After removing sarcomeres of low fluorescence intensity, SarcTrack detected real-time distances between sarcomere domains (eg, Z-discs or M-lines in Figure 1B). The algorithm assumes continuity in the time in which neighboring sarcomeres contract, and the distance between labeled domains, and assesses the maximum and minimum distances between Z-discs (or another sarcomere domain). To detect the duration of contraction and relaxation, individual sarcomeres were each fitted to a custom-designed periodic curve that accounts for asynchronous contraction and relaxation (Figure 1B). Over 200 sarcomeres in each microscopic field of view were imaged to obtain data sets for calculation of specific contractile parameters (Figure 1C).

**Figure 2.**
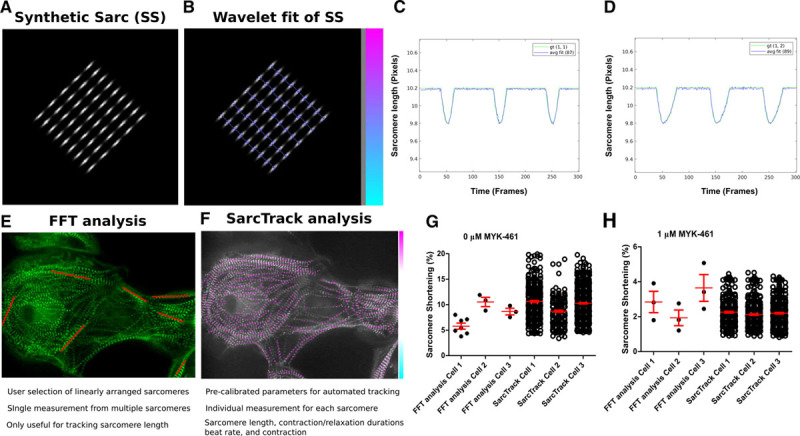
**Comparison of SarcTrack with existing methods of sarcomere tracking and computational benchmarking of SarcTrack with simulated z-disc pairs.** **A**, Simulated sarcomeres produced using a custom MatLab code define adjustable synthetic sarcomeres that can be altered for distances between Z-discs and contraction rates. **B**, Fitting of simulated sarcomeres by SarcTrack showing the goodness of initial fitting using the algorithm, where the colored bar indicates the relative stretch of the wavelet fit with pink being longer sarcomeres and blue shorter. **C** and **D**, This shows ground truth and computed curves of distances for 8 synthetic movies. gt is the ground truth distance curve. The numbers after gt are the proportions of contraction vs relaxation times. For example, gt (1, 2) means relaxation takes twice as long as contraction, and in gt (2, 2) both contraction and relaxation are twice the values in gt (1, 1). The number in parenthesis after avg fit is the number of sarcomeres tracked. The avg fit curves are the average of the curves of distances measured for individual sarcomeres (registered to handle lack of synchronicity). **E**, A frame showing an unpatterned human induced pluripotent stem cell–derived cardiomyocyte (hiPSC-CM) at 100X magnification, where red lines indicate sarcomeres selected for fast Fourier transform (FFT) analysis. FFT analysis tracks the periodicity of many linearly arranged sarcomeres, which is problematic in unpatterned hiPSC-CMs, because of cellular irregularities and nonlinearly arranged sarcomeres. For FFT analyses, the user must manually define regions of interest in each movie, which is inefficient, labor intensive, and can introduce selection bias. FFT provides reliable measures of maximum and minimum sarcomere length, as the signal becomes noisy when aligned sarcomeres number <10–20. **F**, In comparison SarcTrack wavelet labeling of sarcomeres is automated and selects all sarcomeres in a field of view by parametric thresholding as described in Figure 1. SarcTrack labels individual sarcomeres irrespective of orientation and is fully automated once parameters are predefined for a batch of movies. SarcTrack can provide sarcomere length as well as contraction and relaxation durations and beat rate. **G**, Graph showing data analysis of sarcomere shortening from 3 movies that were analyzed by both FFT analysis and SarcTrack analysis. Plotted dots represent a single FFT measure in FFT analysis (derived from red lines shown in **A**) or a single-tracked sarcomere in SarcTrack analysis. SarcTrack provided on average across 3 samples 100-fold more data points, in an automated analysis. **H**, Graph showing the data analysis of sarcomere shortening when cells are treated with 1 μmol/L MYK-461 reducing cellular contractility. No significant differences were observed when the 2 techniques were statistically compared against one another using a Student *t* test where a significance cutoff of *P*<0.05 was used.

To test the fidelity of this system we constructed synthetic computer generated sarcomeres (Figure 2A through 2D). Simulated Z-discs were generated with known pixel distances from their nearest neighbor (Figure 2A; Online Movie I). SarcTrack easily identified the simulated Z-discs (Figure 2B). We simulated asynchronous contraction in 2 different states: (1) initially where contraction and relaxation durations were identical (Figure 2C); or (2) where relaxation was modeled as twice the length as contraction (Figure 2D). The SarcTrack algorithm correctly measured these prescribed contraction and relaxation rates (Figure 2C and 2D).

Additionally, we benchmarked SarcTrack against an FFT method of sarcomere detection (SarcOptiM).^14^ FFT analysis was not intended for nonlinear sarcomeres as exist in hiPSC-CMs, which impeded image analysis (Figure 2E), as only a few rows of sarcomeres (marked in red) could be used in each field of view. In contrast, SarcTrack could reliably mark individual sarcomeres in each field of view irrespective of orientation (Figure 2F). Moreover, the requirement for linear arranged sarcomeres limits the number of samples within a field of view that are appropriate for analyses, which reduces the overall data set. For example, using FFT analyses we measured 4±2 sarcomeres across 3 movies (Figure 2G), and overall 13 sarcomeres with an average contractility of 7.5±0.7%. By contrast, SarcTrack measured 496±160 sarcomeres across these 3 movies and overall 1489 sarcomeres with an average contractility of 10±0.01% (Figure 2G). After applying a compound to decrease cellular contractility (MYK-461), FFT analyses measured a mean of 3 sarcomeres per movie with a mean contractility of 3.2±0.5% (n=9 sarcomeres), whereas SarcTrack measured a mean of 273±35 sarcomeres with a mean contractility of 3.8±0.06% (n=820 sarcomeres; Figure 2H).

**Figure 3.**
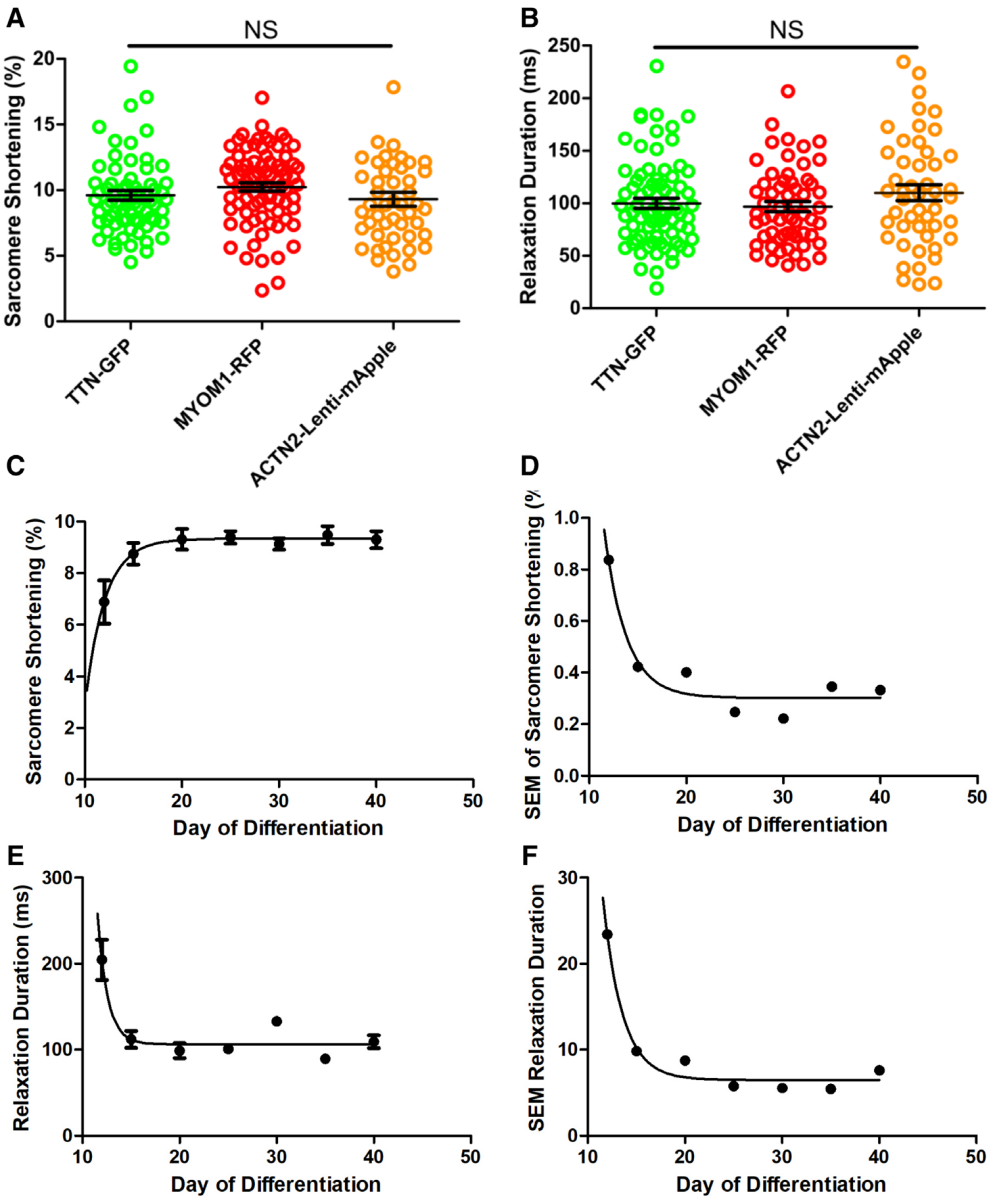
**SarcTrack assessment of sarcomere function in human induced pluripotent stem cell–derived cardiomyocytes (hiPSC-CMs) with different fluorescent tags and throughout differentiation.** **A**, Sarcomere shortening as a percentage of initial sarcomere length, studied at day 30 of differentiation in hiPSC-CM lines with 3 different fluorescent tags labeling the sarcomere Z-disc or M-line. **B**, Relaxation duration (time to peak relaxation after peak contraction) of hiPSC-CM lines. In **A** and **B**, circles denote microscopic field of view, each containing ≈200 sarcomeres. Composite data includes ≈10 000 sarcomeres. There were no statistical differences (denoted as NS) in sarcomere shortening or relaxation durations between hiPSC-CMs with distinct fluorescent tags. **C**, Sarcomere shortening measured in hiPSC-CMs at differentiation days 12 to 40. **D**, The mean SEM of sarcomere contractility as a function of differentiation day. Sarcomere contractility had less variability postdifferentiation day 20 (reduced SEM). **E**, Relaxation duration as a function of differentiation days. Relaxation duration decreased with differentiation day and was stabilized by day 20. **F**, The mean and SEM of relaxation duration decreased with differentiation days and plateaued by day 20. These data infer that hiPSC-CM analyses at day 30 of differentiation should minimize variability. In **C-F**, time points are the average of ≥30 fields of views (each containing 50–300 sarcomeres) from 3 separate differentiations.

To assess whether tagged sarcomeres altered function, we first performed traction stress analyses of TTN-GFP and untagged isogenic hiPSC-CMs (Online Figure VI). There were no significant differences between tagged and untagged hiPSC-CMs. Next, we assessed with the 3 different fluorescent tags altered contractility. SarcTrack analyses of TTN-GFP, MYOM1-RFP, and ACTN-2-mApple, hiPSC-CMs showed comparable contractility and relaxation durations (Figure 3A and 3B; Online Movie II): 9.6±0.3% and 99±4 ms (TTN-GFP); 10±0.3% and 97±4 ms (MYOM1-RFP); and 9.3±0.4% and 109±5 ms (ACTN-2-mApple). Multiple independent differentiations of the TTN-GFP and MYOM1-RFP, hiPSC-CMs were also studied to assess experimental variance among biological replicates. The variance was low across replicates for sarcomere shortening, resting SLs, contraction durations, and relaxation durations (Online Figure VIIA through VIID). The Z-disc labeled TTN-GFP and M-line labeled MYOM1-RFP, hiPSC-CM lines were functionally equivalent for percent contractility and relaxation durations but showed minimal differences (*P*=0.04) in contraction duration and resting SL. Transcriptional analyses of fluorescently tagged and untagged hiPSC-CMs showed no significant differences (Online Figure VIII).

We quantified sarcomere content of hiPSC-CM using SarcTrack to assess maturation during differentiation days 12 to 20. After the initial rapid growth of sarcomeres, the content was stable through day 40 (Table 1). Contractile parameters (Figure 3C through 3F; Table 1) showed that sarcomere shortening increased from 6.9±0.8% at day 12 to a plateau of 9.3±0.4% by day 20. Relaxation duration shortened from 204±23 ms at day 12 to 99±8.7 ms by day 20. By differentiation day 20, contractile parameters plateaued (Figure 3C and 3E), after which there was little variance in these parameters (Figure 3D and 3F).

**Table 1.**
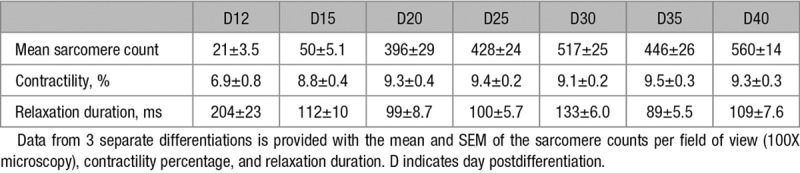
Contractile Parameters Measured Over the Differentiation Time Course

**Figure 4.**
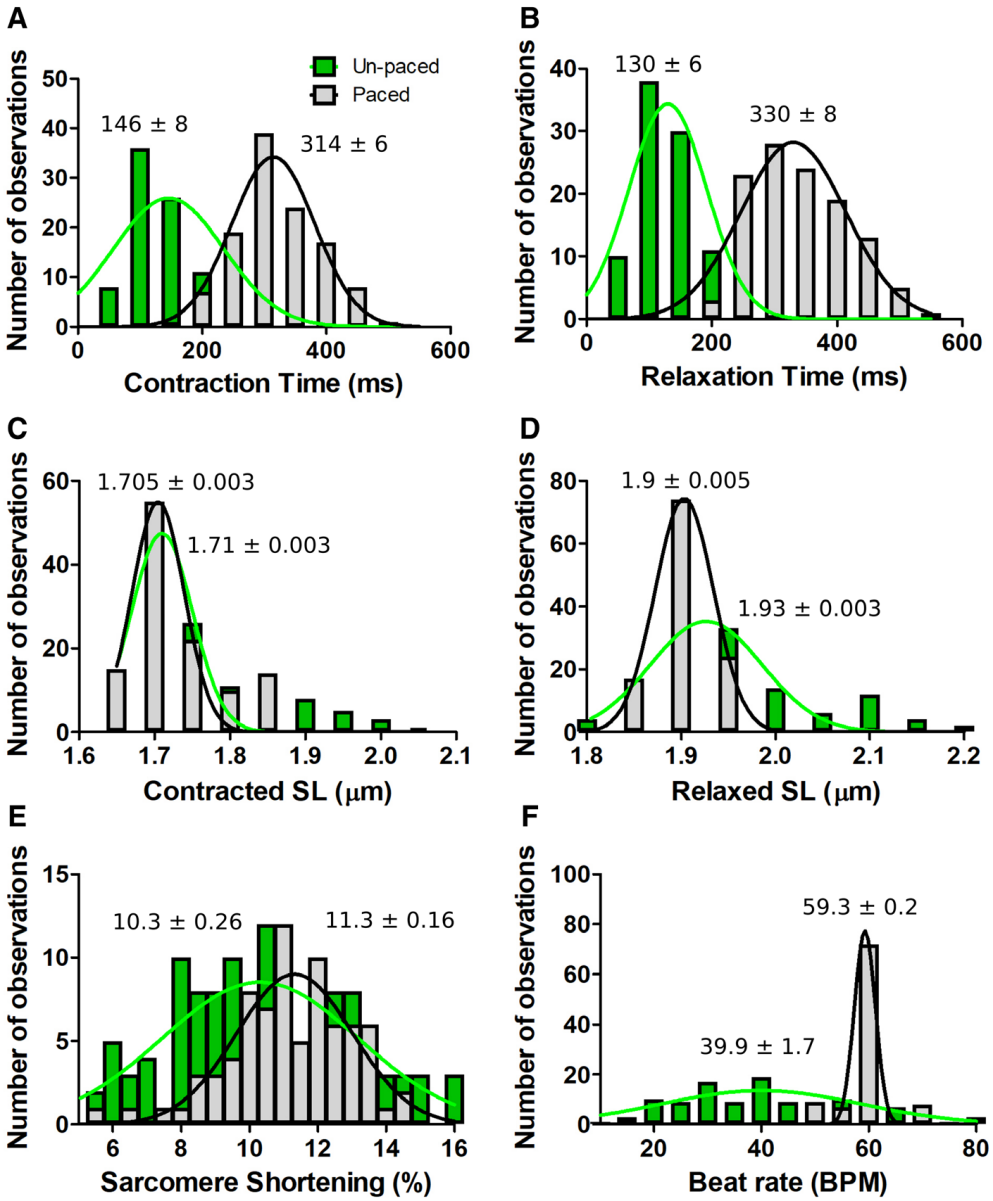
**Effects of electrical pacing on TTN-GFP human induced pluripotent stem cell–derived cardiomyocytes (hiPSC-CMs) contractile parameters assessed by SarcTrack.** **A**, Histogram showing the number of observations as a function of contraction time in paced (gray, 1 Hz stimulation) and unpaced (green) hiPSC-CMs. **B**, Relaxation time as a function of pacing status. **C**, Contracted sarcomere length (SL) as a function of pacing. **D**, Relaxed SL as a function of pacing. **E**, Sarcomere shortening as a function of pacing. **F**, Beat rate as a function of pacing. TTN-GFP hiPSC-CMs were studied at day 30 of differentiation. Observation denotes the mean data from one field of view (≈200 sarcomeres). Histograms were generated from 122 unpaced and 116-paced fields of views. Each histogram was fitted with a single Gaussian distribution and the mean and SEM is indicated.

We also performed parametric analyses of contractile properties in paced and unpaced day 30 TTN-GFP hiPSC-CMs (Figure 4A through 4F; Table 2). Sinusoidal fitting of contractile cycles defined the mean endogenous beat rate of hiPSC-CMs at 39.9±1.7 bpm compared with cells paced at 1 Hz (59.3±0.2 bpm). Beat rate had a substantial impact on contractility parameters. Contraction time (Figure 4A) in unpaced cells was 146±8 ms compared with 314±6 ms in paced cells. Relaxation time (Figure 4B) of unpaced cells was 130±6 ms compared with paced cells 330±8 ms. Beating rate had less influence on contracted SLs than on relaxed SLs (Figure 4C and 4D). Sarcomere shortening was 11.3±0.16% in paced cells and 10.3±0.26% in unpaced cells (Figure 4E).

**Table 2.**
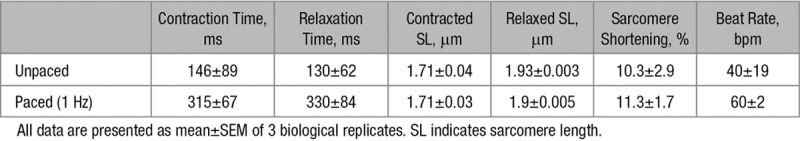
Contractile Parameters Measured as a Comparison of Natural Beat Rate and 1 Hz Pacing

SarcTrack also provided an estimate of the electric adherence to pacing across a cell population (Figure 4F; Online Movie III), and readily identified cells without electric adherence to pacing (typically <5%). Notably, with the exclusion of relaxation times, pacing reduced the SEM for each contractile parameter, thereby highlighting the utility of acute pacing during SarcTrack data acquisition, so as to reduce divergent beat rates among unpaced hiPSC-CMs.

**Figure 5.**
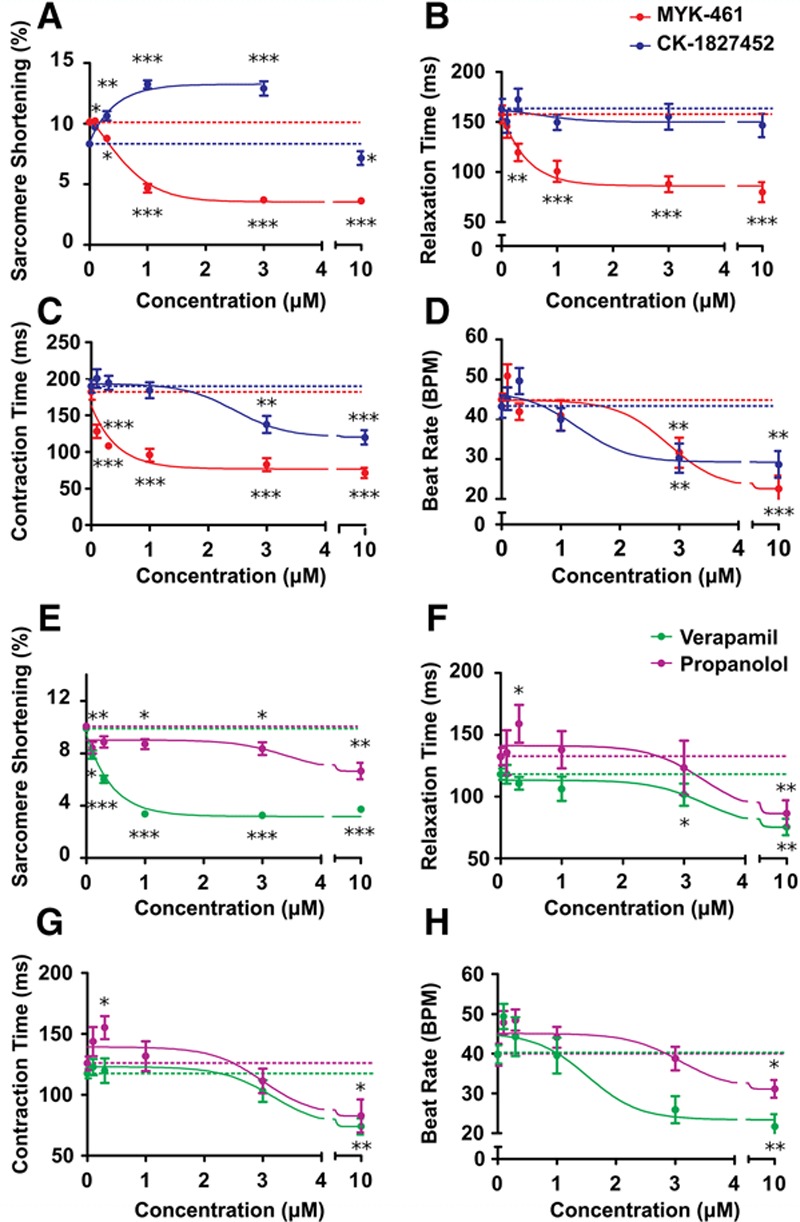
**SarcTrack assessment of contractile effects of pharmacological agents.** **A–D**, Plots of contractile parameters assessed by SarcTrack in day 30 TTN-GFP human induced pluripotent stem cell–derived cardiomyocytes (hiPSC-CMs) varied with dose-dependent increases of CK-1827452 (myosin activator) or MYK-461 (myosin inhibitor). Dotted lines denote the mean of untreated samples, which served as controls. **E–H**, Plots of hiPSC-CMs contractile parameters derived from SarcTrack demonstrate dose-dependent effects of verapamil and propranolol. For each panel, ≈10 fields of view were assessed per drug concentration for each of 3 independent differentiations of hiPSC-CMs. Significances are denoted as *P<0.05, **P<0.01, and ***P<0.001. All values are stated as mean±SEM.

We compared contractility of TTN-GFP hiPSC-CMs after acute exposure to pharmacological agents using SarcTrack (Figure 5A through 5D; Online Figure IX; Online Movies IV and V), including the allosteric myosin ATPase inhibitor, MYK-461^28^ and the myosin activator, CK-1827452. These molecules produced dose-dependent and reciprocal changes in sarcomere shortening (Figure 5A). CK-1827452 caused a dose-dependent increase in contractility from 8.3±0.3% to 12.9±0.6%, except at high dose (10 μmol/L) there was a paradoxical depression (7.2±0.6%), presumably an indication of dose-dependent toxicity. Low concentration (≤2 μmol/L) of CK-1827452 had little effect on the duration of contraction and relaxation but high doses decreased the duration of contraction (Figure 5B and 5C). MYK-461 decreased sarcomere shortening and increased the duration of both contraction and relaxation in hiPSC-CMs. Both CK-1827452 and MYK-461 reduced endogenous beating rates (Figure 5D).

Parallel analyses of commonly used cardiac drugs that indirectly modulate cardiomyocyte contractility showed distinct effects on sarcomere performance (Figure 5E through 5H). Propranolol, which targets both β_1_ and β_2_ adrenergic receptors, reduced beat rates similar to CK-1827452 but also reduced sarcomere shortening, with minimal effects on durations of contraction and relaxation, except at high doses. Verapamil, which blocks voltage-dependent calcium channels, reduced sarcomere shortening as did MYK-461, but with minimal effects on the durations of contraction and relaxation, except at high doses.

**Figure 6.**
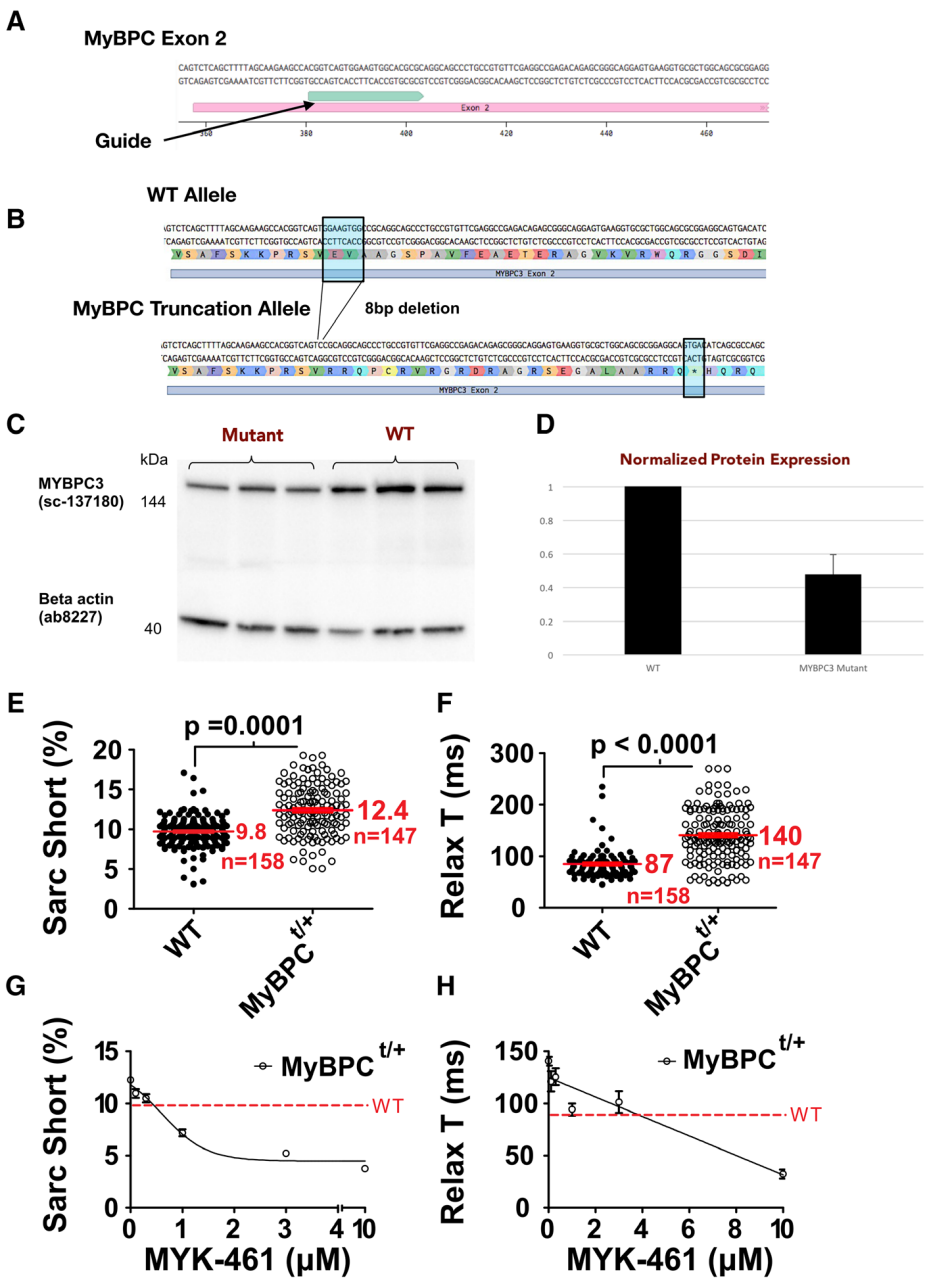
**MyBPC (myosin-binding protein C) haploinsufficiency modeled in TTN-GFP human induced pluripotent stem cell–derived cardiomyocytes (hiPSC-CMs) and studied by SarcTrack.** **A**, Guide RNA used to target exon-2 of *MYBPC3* in TTN-GFP hiPSC-CMs for CRISPR/Cas9 mutagenesis. **B**, Schematic representation of the excision of 8 nucleotides resulting in a premature stop codon (*) in exon-2. **C**, Western blot of MyBPC protein and β-actin obtained from 3 independent differentiations of wild-type (WT) and MyBPC mutant TTN-GFP hiPSC-CMs at differentiation day 30. **D**, MyBPC protein normalized to β-actin expression in WT and MyBPC mutant hiPSCs demonstrates 50% reduction of MyBPC protein content. **E** and **F**, SarcTrack analyses of heterozygous MyBPC^t/+^ TTN-GFP hiPSC-CMs and isogenic controls (nucleofected with CRISPR-Cas9 but without *MYBCP*3 guides) showed indistinguishable beat rates of 36±1.4 ms, but MyBPC^t/+^ TTN-GFP hiPSC-CMs had increased sarcomere contractility and prolonged relaxation. **G** and **H**, The allosteric myosin inhibitor MYK-461 caused dose-dependent reduction in contractility and improved relaxation in MyBPC^t/+^ TTN-GFP hiPSC-CMs, highlighting SarcTrack as an effective platform for assessing the parametric effects of compounds on cardiomyocyte contractility.

To determine whether SarcTrack could detect the effects of human genetic variants, we engineered a heterozygous truncation of myosin-binding protein C (MyBPC^t/+^; Figure 6A and 6B) into TTN-GFP hiPSCs. This variant recapitulates the genotype of many patients with hypertrophic cardiomyopathy (HCM).^29,30^ After differentiation into hiPSC-CMs, MyBPC (myosin-binding protein C) protein levels were reduced by 44% (*P*=0.01) of normal (Figure 6C and 6D), demonstrating haploinsufficiency. SarcTrack data from day 30 isogenic wild-type and MyBPC^t/+^ hiPSC-CMs, across multiple differentiations (Figure 6E through 6H; Online Movie VI), demonstrated that the HCM variant increased cellular shortening (12.4% versus 9.8%; *P*=0.0001) and slowed relaxation (140 ms versus 87 ms; *P*<0.0001). These in vitro phenotypes recapitulate the human cardiac manifestations of HCM: hypercontractility and impaired relaxation.^29,31^ MYK-461 treatment of mutant hiPSC-CMs reversed these abnormalities, restoring function comparable to in vivo responses observed in HCM mice carrying myosin mutations^28^ (Figure 6G and 6H). SarcTrack data indicated that low dose (1 μmol/L) MYK-461 normalized hypercontractility, whereas higher doses (2–4 μmol/L) were needed to normalize relaxation.

## Discussion

We demonstrate that SarcTrack, a MatLab algorithm, efficiently measures contractile parameters of hundreds of individual sarcomeres from a single movie, and enables rapid evaluation of sarcomere function from a single 5-second movie. The algorithm is optimized to handle nonlinearly arranged sarcomeres in hiPSC-CMs, as each sarcomere is independently assessed. By capturing multiple videos and fields of view, the method simultaneously provides a data set that includes thousands of sarcomere measurements per experimental condition—a scale of data that has never been achieved with previous methodologies that assess sarcomere performance. SarcTrack is adaptable for analyses of sarcomere and muscle physiology, studies of human gene variants, and assessments of both desirable and unintended effects of pharmacological agents on contractility.

The underlying technique used in SarcTrack is a simulated sarcomere, which we defined by a set of 6 parameters and then iteratively fitted to each individual sarcomere in a video frame. The algorithm tracked changes in SL throughout each frame of a movie to define sarcomere displacement, and provided measures of peak contracted and peak relaxed SLs, percent contraction, relaxation duration, contraction duration, and beat rate for each sarcomere. Users define and test settings for sarcomere fitting (as described in Online Methods), after which subsequent analyses are fully automated. Computational processing of each video requires on 15 minutes and occurs without user input and once movie data are collected, downstream analyses are rapid, unbiased, and user-friendly. SarcTrack software can be combined with the plethora of existing methodologies used to visualize sarcomeres, so that the algorithm can be widely applicable to researchers from multiple disciplines of muscle biology.

We benchmarked SarcTrack by studying synthetic sarcomeres with asymmetrical contraction and compared it with an existing FFT analysis platform.^14^ We demonstrated that SarcTrack provided superior accuracy in measured contractility of Z-disc pairs and more robust and rapid functional assessment of sarcomeres in hiPSC-CMs.

Using SarcTrack, we showed that the contractile parameters in hiPSC-CMs with different fluorescent tags are indistinguishable, and we provided functional data during stages of hiPSC-CM differentiation and maturation. In response to pharmacological perturbations that directly (CK-1827452 and MYK-461) or indirectly (verapamil and propranolol) modulated sarcomere function, we observed therapeutic and cardiotoxic effects on contractile parameters. SarcTrack analyses of hiPSC-CMs with a heterozygous truncation of MyBPC revealed prototypic clinical manifestations of human HCM.^31^ Application of MYK-461 to mutant hiPSC-CMs abated these phenotypes, evidencing that the potential therapeutic efficacy of this molecule in human HCM patients.

Limitations in the SarcTrack platform included the need for fluorescent labeled, paired domains of sarcomeres in cells without high levels of background signal. Fluorescent labeling allows precise tracking of individual sarcomeres, at far higher resolution than can be achieved by bright-field techniques.^11,12^ However, if fluorescent labeling is not feasible, the SarcTrack algorithm could be trained to define sarcomere periodicity using second harmonics originating from the myosin rod α-helix, which can be assessed using second harmonic generation microscopy. Input videos must provide ≥30 frames per second with good signal-to-noise ratio. We used a relatively low frame rate of 30 frames per second to demonstrate that the algorithm can be applied in conjunction with fluorescence confocal microscopes that are not capable of obtaining high frame rate videos, thus extending the utility of this algorithm to those that do not possess more expensive imaging modalities. SarcTrack code can sequentially analyze individual videos on a personalized computer, however, researchers may prefer to perform simultaneous analyses of 4 or more videos, which may require a dedicated computational hub. Finally, as with all cell-based platforms, the viability and overall health of cells can impact data. Despite these issues, the level of precision achieved for analyzing sarcomere dynamics and the versatility to multiplex this platform with existing technologies such as traction force microscopy,^4^ multidimensional patterning techniques,^32,33^ and engineered multicellular tissues^5,7^ allows SarcTrack to define deeper insights into contractile mechanics including subtle effects on sarcomere structure and contractility in response to genetic variants and small molecules.

In summation, SarcTrack enables rapid evaluation of sarcomere contractile function. The platform is optimized for hiPSC-CMs but can be readily adapted to other myocyte types. With modern cardiovascular research transitioning from basic mechanistic discoveries to therapeutic applications, we expect that novel method such as SarcTrack will further accelerate translational cardiovascular medicine.

## Acknowledgments

We thank the members of the Image and Data Analysis Code at Harvard Medical School (HMS) for their discussions and technical assistance with programming SarcTrack. We thank the members of the HMS computing core for their help running SarcTrack and implementation on the HMS computing cluster.

## Sources of Funding

Support for this study was provided in part by the Wellcome Trust (C.N.T.: Sir Henry Wellcome fellowship), the Fondation Leducq (J.G.S., C.E.S., and fellowship to M.M.), the German Academic Scholarship Foundation (M.N. and M.S.), the Konrad-Adenauer Foundation (M.M.), the Engineering Research Centers Program of the National Science Foundation (A.C., C.S.C., J.G.S., and C.E.S.: NSF Cooperative Agreement number EEC-1647837), the National Institutes of Health (J.R. and O.P.: 5R01HD085121; J.G.S.: U01HL098166 and fellowship to A.S.; and J.G.S. and C.E.S.: 5R01HL080494 and 5R01HL084553), and the Howard Hughes Medical Institute (C.E.S.).

## Disclosures

C.E.S. and J.G.S. are founders and own shares in MyoKardia Inc, a start-up company that is developing therapeutics that target the sarcomere. MyoKardia was not involved in any aspect of this study. The other authors report no conflicts.

## Supplementary Material

## Novelty and Significance

### What Is Known?

- Many cardiovascular diseases and pharmacological agents alter cellular contractility.
- Human induced pluripotent stem cell–derived cardiomyocytes provide a human model for screening variants that may cause disease and compounds that may alter cardiomyocyte functions.
- Tools are limited that provide automated, high-fidelity, and direct assessments of sarcomeres, the contractile unit within human induced pluripotent stem cell–derived cardiomyocytes.
