# Supplementary material for: SarcTrack: An Adaptable Software Tool for Efficient Large-Scale Analysis of Sarcomere Function in hiPSC-Cardiomyocytes
Source: Circ Res. 2019 Apr 11;124(8):1172–83. doi: 10.1161/CIRCRESAHA.118.314505 (PMC6485312; doi:10.1161/CIRCRESAHA.118.314505)
Supplement: Supplementary file 1 [file res-124-1172-s001.pdf]

Online Methods

Manual for Running SarcTrack

Download all files at the following link :https://github.com/HMS-IDAC/SarcTrack.

Step 1: DWProcessFrame

Once downloaded open MatLab and select dwProcessFrame, this part of the algorithm tests user defined parameters on a single frame of an acquired image stack. You will need to define the .avi file location on your computer at the top of this section as in the image below: path = '/Users/……

One can then specify the frame from the selected movie that should be tested: frameIndex = 0 is the first frame of the movie, frameIndex = 1 would be the second frame of the movie.

```
clear, clc

%% parameters

path = '/Users/Username/Desktop/trial/1.avi';
frameIndex = 0;

ds = 5:0.2:12; % range of distances (in pixels)
stretch = 1; % stretch of morlet wavelet
scale = 1; % scale of morlet wavelet
nangs = 8;
hopsize = 8;
halfwindowsize = 4;
magthreshold = [];
convtype = 'real+';

%% read frame, show wavelets

v = VideoReader(path);
v.CurrentTime = frameIndex/v.FrameRate;
frame = readFrame(v);
I = double(rgb2gray(frame))/255;
% imshow(I)

mr1 = normalize(smorlet2(ds(1),stretch,scale,90));
mr2 = normalize(smorlet2(ds(end),stretch,scale,90));
J = normalize(I);
```

Below this there are 6 definable parameters that SarcTrack uses to fit the double wavelet to the sarcomeres in each frame of the image stack. These will differ depending on the users µm/pixel count. Below we give approximate starting values for a movie stack acquired at 960x720 with 0.15 µm/pixel. The user must define these parameters they should be fine-tuned as below:

> **1: ds** = (Distance between wavelet peaks) 5:0.2:12: minimum pixel distance, steps between in pixels, and maximum pixel distance. As these are all defined in pixels they will depend on the resolution of the images in the users image stack.
>
> E.g. set an upper limit of sarcomere length this prevents SarcTrack from trying to fit a wavelet pair over multiple sarcomeres. We usually use the equivalent pixel length that corresponds to 2.4 µm or above (15 pixels). This avoids fitting of the wavelet pair over two sarcomeres. We also set a minimum pixel length that corresponds to 1.4 µm (9 pixels) as this is the theoretical smallest sarcomere length, as thick filaments themselves are somewhere between 1.4-1.6 µm in length. This can be iteratively fine-tuned by the user once

dwProcessFrame has been run and checked by eye. We usually allow the wavelets to fit every 0.2 pixels between 1.4-2.2 e.g. 1.42,1.44,1.46…. and so on to 2.2. Adding smaller fit periodicities increases analysis time as it allows further degrees of freedom for the wavelet fitting to be analyzed.

2: Stretch (Elongation) of each wavelet this is the constrained length of each wavelet (e) in Figure 1A. This is an adjustable value. A typical value we would start with at an image size of 960x720 with 0.15 microns per pixel is 1.

3: Scale (sigma), which defines the width of each individual wavelet of the pair and for an image of dimensions 960x720 at 100X with 0.15 μm/pixel we have typically used 1.

4: nangs (alpha) the number of angles the wavelet pair can test for best fitting to z-disc pairs, for instance entering a value of nangs of 8 would allow the wavelet to search every 22.5^0 from 0 -180^0. The more degrees of freedom the wavelet pair has for fitting the longer analysis will take but it is often worth trying a starting value of 8. If this does not suffice the user can set higher values, this will increase analysis time as the wavelet pair has further degrees of freedom to search for a best fit.

5: hopsize (pixels) the distance between gridpoints, we usually use 16 pixels, i.e. we shouldn't find more than one sarcomere within a box of dimensions 16 x 16 pixels (2.4 x 2.4 μm), this is done to avoid re-fitting the same sarcomere multiple times.

6: Halfwindowsize (pixels) the neighbourhood around each grid where the best fit for the wavelet pair can be approximated. This value should be below half of the pixel size used for hopsize i.e. using 16 for hopsize use Halfwindowsize < 8.

Once these parameters have been set analysis can begin. You will find a run button that allows the script to begin running. This process will return back to the user a single frame showing how wavelet pairs have been fit (As shown in Figure 1A). After review by the user, the parameters listed above can be adjusted. This step should be repeated until wavelet pair fitting are coincident with the majority of sarcomere in a microscopic field of fiew .

Step 2: dwProcessFolder on a single movie

Double-click dwProcessFolder and provide the file extension of a folder containing a single .avi movie as described above. The script will copy the parameters set in dwProcessFrame that approximated sarcomere fitting and apply them to the movie.

Now dwProcessFolder can be run with these pre-defined parameters, the script will perform the same fitting that was performed in step 1 to every frame in the .avi file.

As defined in Figure 1B this will provide a set of output files, three .csv files with raw data and a folder with all of the frames fit with the double wavelet. These files have values that are all in raw values of pixels and frames. To scrutinize these outputs the user can run dwCheckResults simply copy the folder location in 'path =' in dwCheckResults this will populate three graphical outputs from the three .csv files, as shown in Figure 1B.

Step 3: dwProcessFolder on batch movies

Once fidelity of the single movie has been checked one can now move multiple movies to a single folder and re-run dwProcessFolder to analyze all movies in a specific folder location.

This will provide all three output .csv files and a folder of fit frames for each movie. These outputs can then be adjusted in excel to µm and seconds using the appropriate frame/second and µm/pixel count for the users acquisitions. Alternatively, the user can apply our own conversion and collating scripts ‘wheres_that_sarcomere.R’ and ‘make_ave_table1.sh’. ‘wheres_that_sarcomere.R’ converts all values to seconds and µm. The user will need to add their own conversion values for pixels and frames in the lines 9-13. ‘wheres_that_sarcomere.R’ will calculate an average and standard deviation for each parameter from each movie and collate them into a single row in a final .csv file.

ONLINE FIGURES

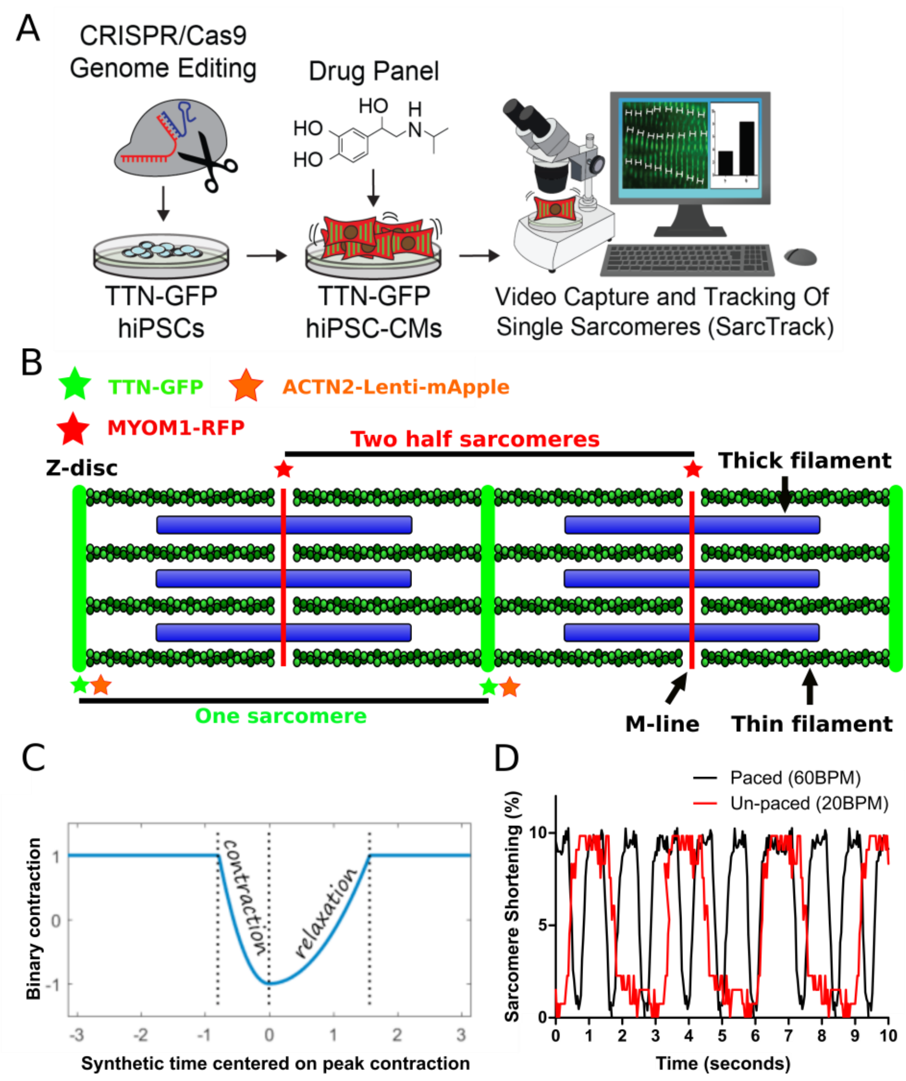

Online Figure I: A workflow for using SarcTrack to measure sarcomere contractility in fluorescent sarcomere reporter hiPSC-CM lines. **A)** CRISPR/Cas9 technology yields fluorescently tagged hiPSCs that can be further genome-edited and/or probed with pharmacologic agents for functional assessment using SarcTrack. **B)** Schematic of two sarcomeres approximating the location of fluorescent tags used for SarcTrack analysis, using distinct methodologies of labelling sarcomere proteins (CRISPR/Cas9-mediated genome editing and lentiviral transfection). **C)** Illustration of contraction and relaxation times using a synthetically generated contraction. **D)** Contractile cycles of a paced (60 bpm) sarcomere (black line) compared to a spontaneously beating (20 bpm) sarcomere (red line).

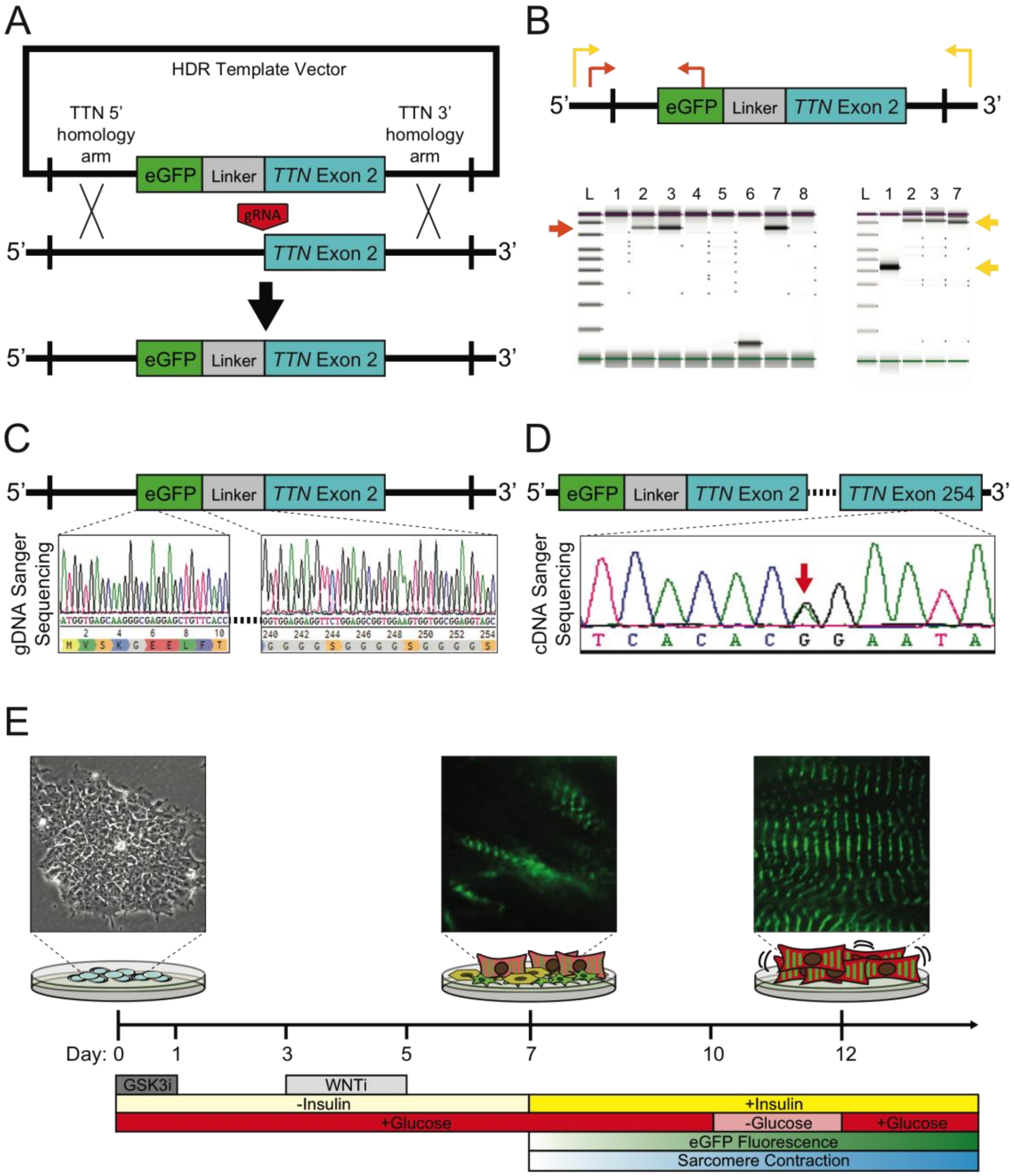

Online Figure II: Generation and differentiation of the TTN-GFP hiPSC reporter line. A) Template vector design for the insertion of eGFP with flexible linker into the *TTN* Exon 2. **B)** Primer design for testing the insertion of the eGFP and linker onto *TTN* exon 2. **C)** Sanger sequencing confirming the insertion of the eGFP and linker adjacent to *TTN* exon 2. **D)** A single nucleotide polymorphism in the exon 254 of TTN-GFP cDNA confirmed the expression of both *TTN* alleles. **E)** Differentiation of the TTN-GFP reporter line showing the assembly of sarcomeres over the differentiation time course, as well as the protocol used for differentiation.

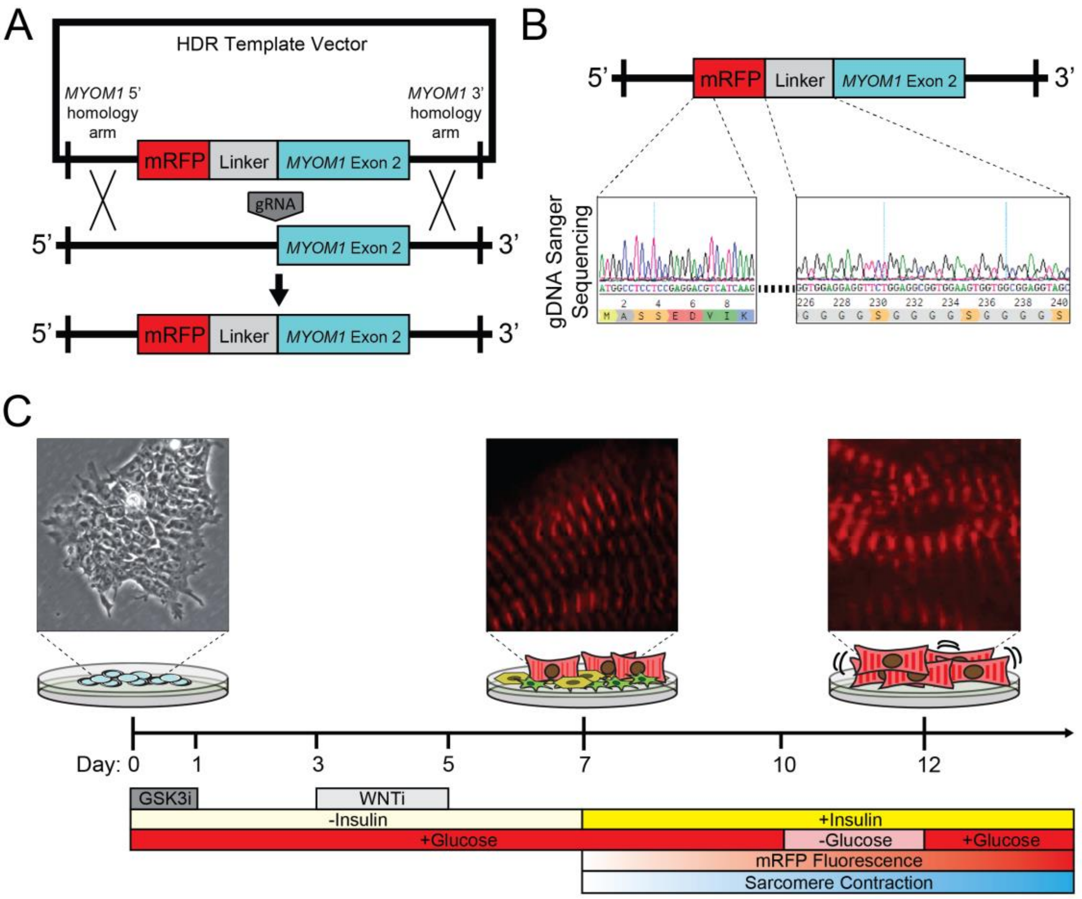

Online Figure III: Generation and differentiation of the MYOM1-RFP hiPSC reporter line. A) Diagram of template vector for homology directed repair to generate the MYOM1-RFP line. **B)** Insertion site of mRFP and flexible linker into *MYOM1* exon 2 with Sanger validation of insertion. **C)** Pictorial timeline and protocol of MYOM1-RFP hiPSC differentiation to produce beating hiPSC-CMs.

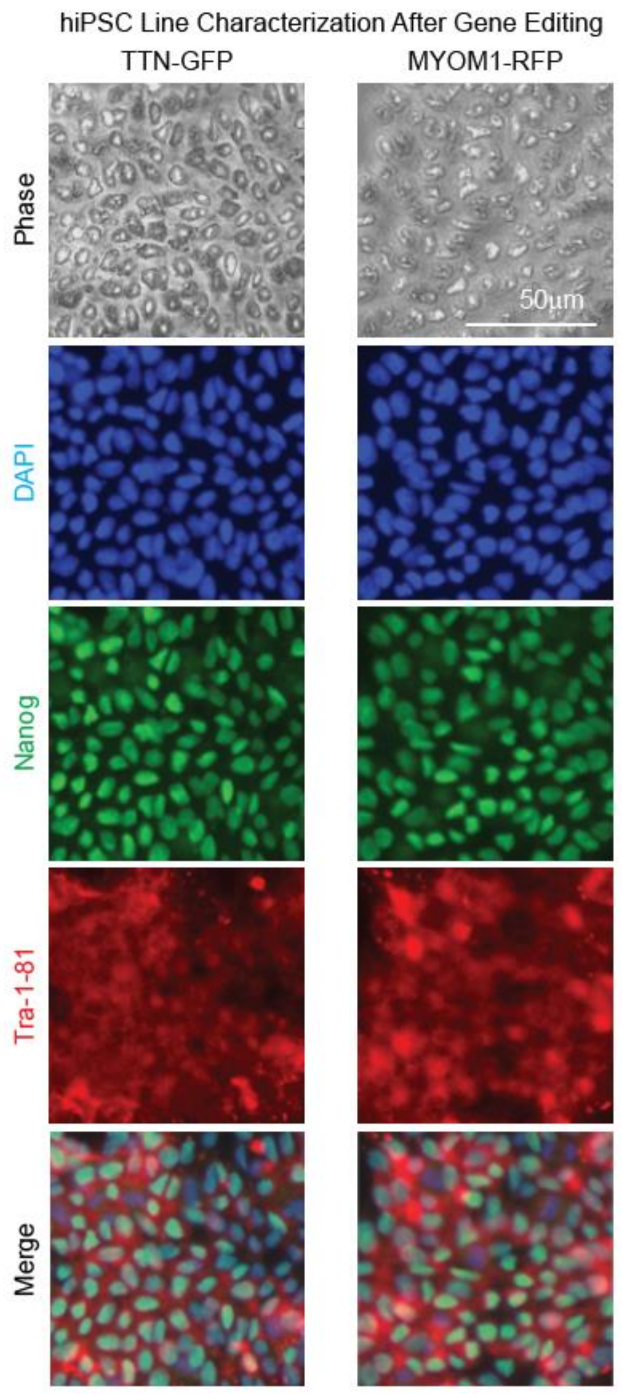

Online Figure IV: Characterization of fluorescent sarcomere reporter hiPSC lines. Immunofluorescence-based characterization of TTN-GFP and MYOM1-RFP hiPSC lines after generation via CRISPR/Cas9 gene editing. The hiPSCs from all lines expressed standard pluripotency markers Nanog and Tra-1-81 and exhibited a standard, tightly-packed hiPSC morphology. Immunostaining was performed using standard procedures with 4% Paraformaldehyde fixation, TX-100 membrane permeabilization, bovine serum albumin for blocking, primary antibodies against Tra-1-81 (Millipore MAB4381, 1:100) and Nanog (Abcam ab80892, 1:100), and secondary antibodies with conjugated fluorophores (Abcam).

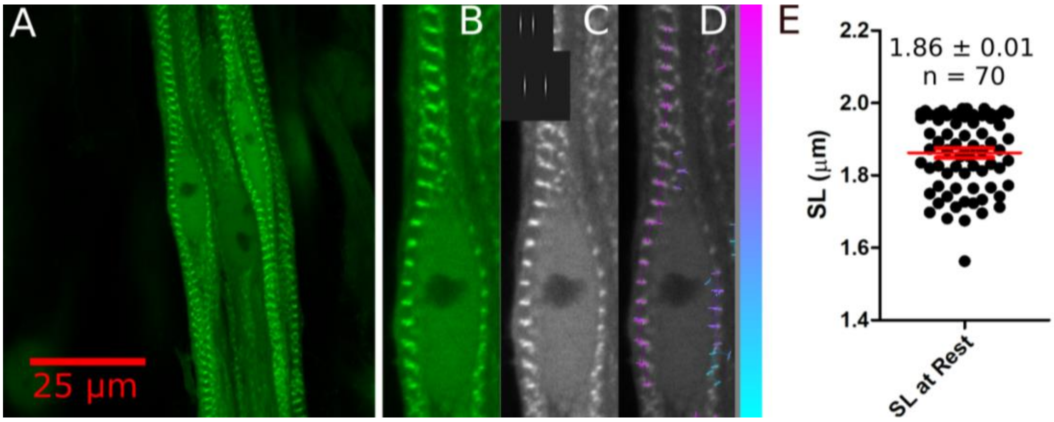

Online Figure V: Use of SarcTrack on TTN-GFP hiPSC-derived skeletal muscle cells. **A)** 60X image of skeletal muscle fibers derived from hiPSCs with an endogenous TTN-GFP tag. **B)** Sections demonstrate clear striations due to Z-disc labelling by TTN-GFP. **C)** Grey scale image with inset wavelet detect the smallest and largest sarcomere lengths. **D)** Fitting of wavelets to Z-discs showing sarcomere lengths. **E)** Plot of sarcomere lengths from image A showing the applicability of SarcTrack to assess contractility in skeletal muscle fibers derived from the TTN-GFP hiPSC line.

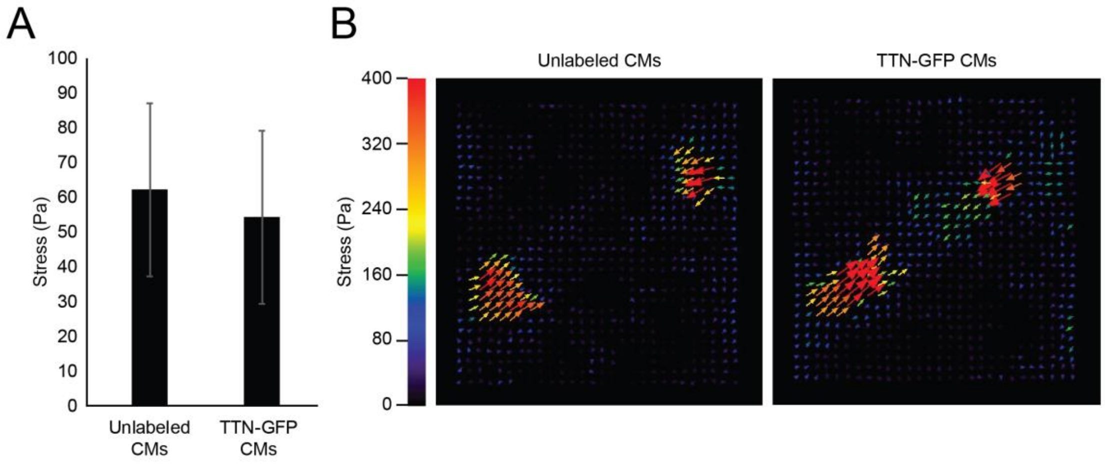

Online Figure VI: Traction stress measurements of single iPSC-CMs show no functional difference between unlabelled and TTN-GFP labelled CMs. A) Bar plot of Stress (Pa) generated by iPSC-CMs on 7.9 KPa PAA gels indicating no significant difference between unlabelled (n = 12) and TTN-GFP labelled CMs. (n = 9) **B)** Representative heat-maps showing raw traction stress of both unlabelled and labelled CMs.

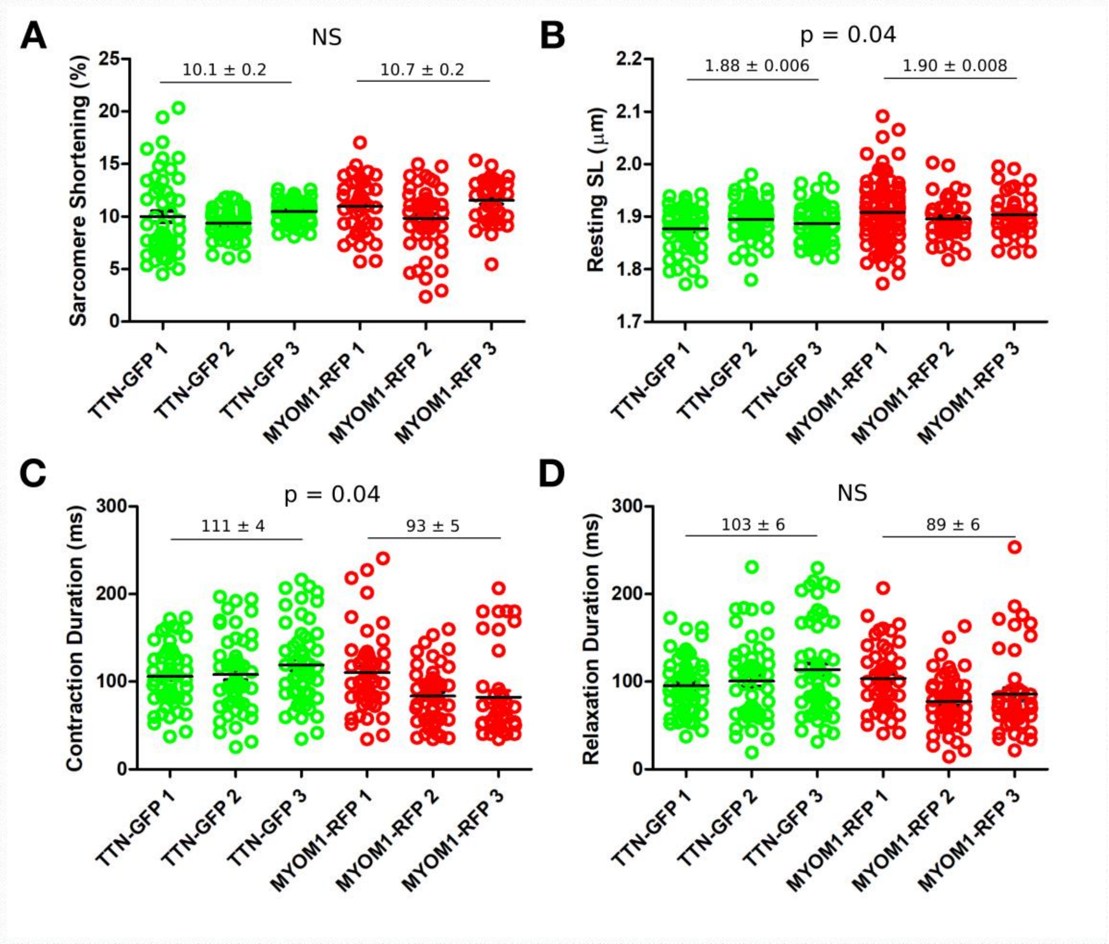

Online Figure VII: SarcTrack-based functional comparison of independent differentiations for TTN-GFP and MYOM1-RFP reporter hiPSC-CM lines at day 30. A) Sarcomere shortening was assessed by SarcTrack across three separate differentiations of TTN-GFP and MYOM1-RFP day 30 hiPSC-CMs. **B)** Resting sarcomere length in day 30 hiPSC-CMs assessed by SarcTrack. **C)** Contraction duration in day 30 hiPSC-CMs assessed by SarcTrack. **D)** Relaxation duration assessed in day 30 hiPSC-CMs by SarcTrack. All means across multiple differentiations are stated with p values between TTN-GFP and MYOM1-RFP indicated above each plot.

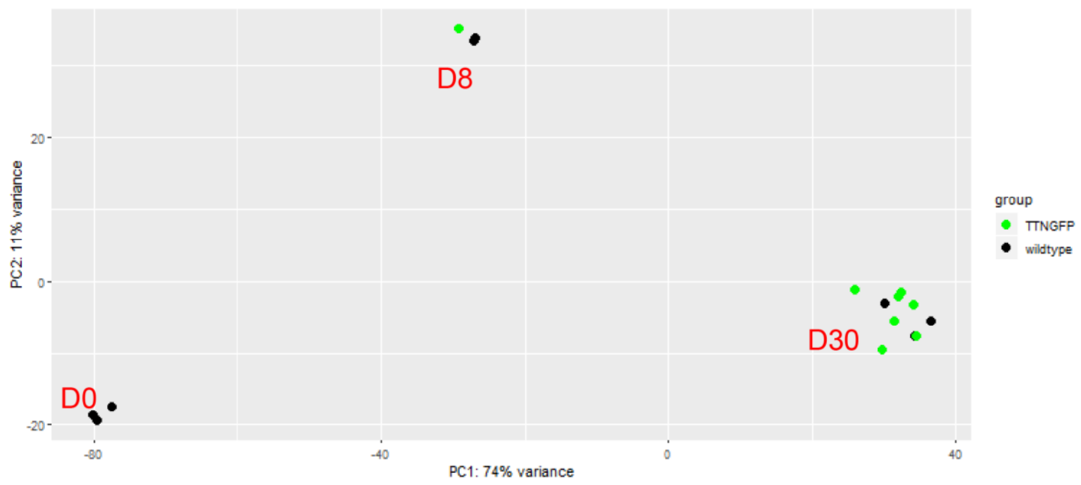

Online Figure VIII: Gene expression principal component analysis of wild type vs TTNGFP hiPSC-CMs shows minimal difference in gene expression profile between genotypes. Gene expression principal component analysis (PCA) of RNA-sequencing for day 0, 8, and 30 PGP1 WT and TTNGFP hiPSC-CMs. Every dot represents an independent biological replicate differentiation.

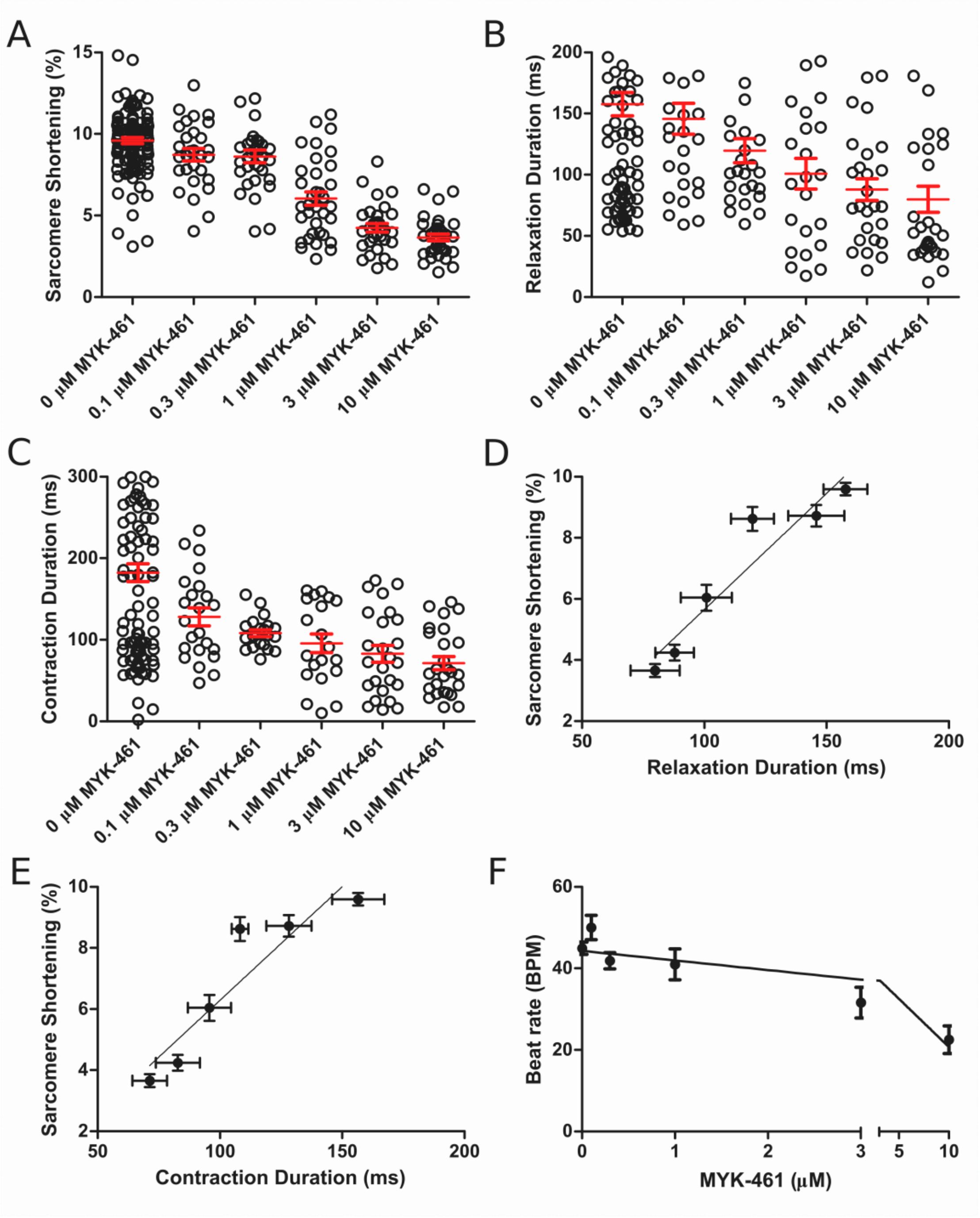

Online Figure IX: Raw data of MYK-461 effect on individual fields of view assessed by SarcTrack in TTN-GFP hiPSC-CMs. A-C) Each field of view plotted for specified concentration of drug used to treat day 30 TTN-GFP hiPSC-CMs, showing the data spread per dataset when averaging field of views. Each field of view sampled on average ~200 sarcomeres. **D-E)** Sarcomere shortening as a function of relaxation duration or contraction duration, assessed by SarcTrack, showed a strong positive correlation observed across MYK-461 concentrations.

SUPPLEMENTAL MOVIES:

Online Movie I: Synthetic sarcomere beating asynchronously.

Online Movie II: Representative movies of TTN-GFP, MYOM1-RFP and ACTN2-Lenti-mApple hiPSC-CMs spontaneously beating.

Online Movie III: Representative movie of TTN-GFP hiPSC-CMs imaged beating spontaneously and paced at 1Hz.

Online Movie IV: Movie of TTN-GFP hiPSC-CMs either untreated or treated with myosin activator CK-1827452 (0.1-10 μM).

Online Movie V: Movie of TTN-GFP hiPSC-CMs either untreated or treated with myosin ATP-ase inhibitor MYK-461 (0.1-10 μM).

Online Movie VI: Comparison of TTN-GFP WT hiPSC-CMs with TTN-GFP MyBPC$^{t/+}$ hiPSC-CMs.
